# Speech silence character as a diagnostic biomarker of early cognitive decline and its functional mechanism: a multicenter cross-sectional cohort study

**DOI:** 10.1186/s12916-022-02584-x

**Published:** 2022-11-07

**Authors:** Hua-Long Wang, Ran Tang, Ru-Jing Ren, Eric B. Dammer, Qi-Hao Guo, Guo-Ping Peng, Hai-Lun Cui, You-Min Zhang, Jin-Tao Wang, Xin-Yi Xie, Qiang Huang, Jian-Ping Li, Fu-Hua Yan, Sheng-Di Chen, Na-Ying He, Gang Wang

**Affiliations:** 1 Department of Neurology and Institute of Neurology, Ruijin Hospital, Shanghai Jiao Tong University School of Medicine, Shanghai, 200025 People’s Republic of China; 2 Department of Neurology, The First Hospital of Hebei Medical University; Brain Aging and Cognitive Neuroscience Laboratory of Hebei Province, Shijiazhuang, 050031 Hebei People’s Republic of China; 3 Department of Biochemistry and Center for Neurodegenerative Disease, Emory University School of Medicine, Atlanta, GA 30322 USA; 4 Department of Gerontology, Shanghai Jiao Tong University Affiliated Sixth People’s Hospital, Shanghai, People’s Republic of China; 5 Department of Neurology, The First Affiliated Hospital, Zhejiang University School of Medicine, Hangzhou, People’s Republic of China; 6 Department of Radiology, Ruijin Hospital, Shanghai Jiao Tong University School of Medicine, Shanghai, People’s Republic of China

**Keywords:** Alzheimer’s disease, Amnestic mild cognitive impairment, Percentage of silence duration, Functional MRI

## Abstract

**Background:**

Language deficits frequently occur during the prodromal stages of Alzheimer’s disease (AD). However, the characteristics of linguistic impairment and its underlying mechanism(s) remain to be explored for the early diagnosis of AD.

**Methods:**

The percentage of silence duration (PSD) of 324 subjects was analyzed, including patients with AD, amnestic mild cognitive impairment (aMCI), and normal controls (NC) recruited from the China multi-center cohort, and the diagnostic efficiency was replicated from the Pitt center cohort. Furthermore, the specific language network involved in the fragmented speech was analyzed using task-based functional magnetic resonance.

**Results:**

In the China cohort, PSD increased significantly in aMCI and AD patients. The area under the curve of the receiver operating characteristic curves is 0.74, 0.84, and 0.80 in the classification of NC/aMCI, NC/AD, and NC/aMCI+AD. In the Pitt center cohort, PSD was verified as a reliable diagnosis biomarker to differentiate mild AD patients from NC. Next, in response to fluency tasks, clusters in the bilateral inferior frontal gyrus, precentral gyrus, left inferior temporal gyrus, and inferior parietal lobule deactivated markedly in the aMCI/AD group (cluster-level *P* < 0.05, family-wise error (FWE) corrected). In the patient group (AD+aMCI), higher activation level of the right pars triangularis was associated with higher PSD in in both semantic and phonemic tasks.

**Conclusions:**

PSD is a reliable diagnostic biomarker for the early stage of AD and aMCI. At as early as aMCI phase, the brain response to fluency tasks was inhibited markedly, partly explaining why PSD was elevated simultaneously.

**Supplementary Information:**

The online version contains supplementary material available at 10.1186/s12916-022-02584-x.

## Introduction

Alzheimer’s disease (AD) is the most common neurocognitive disorder, with memory deficits being the earliest and most characteristic symptom, and this is accompanied by other cognitive deficits such as executive dysfunction, apraxia, and aphasia [1]. In the past few decades, major progress has been made in the development of biofluid or neuroimaging biomarkers for AD diagnosis, such as cerebrospinal fluid measures and in situ imaging of Aβ and phosphorylated tau, other neuroimaging techniques, and neuropsychological tests [1]. However, these methods are limited by their high cost and invasive nature.

Language deficits are detected from the prodromal stages of AD or amnestic mild cognitive impairment (aMCI) and have been considered as a candidate biomarker for early diagnosis [2–4]. Most of these studies focus either on identifying characteristic linguistic parameters or using them to discriminate between healthy older people and those affected by aMCI or AD, and they indicate a large number of language components with ideal diagnostic values for discriminating AD, yet results were heterogeneous due to the variety of methods and vocal features being examined [5], not to mention the potential influences of distinct language spoken by subjects, or even the dialects of a particular language. And no single biomarker accurately diagnoses all cases of AD. Among them, pauses are often investigated as a hallmark of the lexical-semantic decline during speech production in AD [6, 7] and may be the key factor corresponding to speech fluency which is mainly determined by semantic and phonemic fluency [8].

Given that silent pauses are involved with impairment in multiple cognitive abilities, e.g., word retrieval, working memory, and execution, we put emphasis on the most important aspect—lexical-semantic processing and its functional alteration in AD or aMCI [9]. Further, the pause frequency in picture-based narrative has been reported to be associated with verbal fluency and grey matter density of anterior temporal lobe [2, 6, 10]. Although the task-based functional magnetic resonance imaging (fMRI) technique is a popular method to visualize brain areas supporting specific cognitive stimuli [11], to date, a limited number of studies have focused on functional alteration on the language network and its relationship with silent pauses.

Our previous study suggested that the computer-based analysis of certain language components could be a promising diagnostic method for early AD and aMCI [2] and highlighted the application of percent silence duration (PSD, in which silence is defined as the summed duration of all silent segments of the recording, mainly the various pauses) as a potentially reliable biomarker for the early stage of cognitive decline due to AD with translingual diagnostic value. In order to fully determine the translingual diagnostic value of PSD and its related brain network alteration, we will confirm the diagnostic value of PSD in the Chinese multi-center cohort and further validate its diagnostic value in an English-speaking cohort from the Pitt database, and the brain networks involved in verbal fluency which are related to PSD using a task-based fMRI experiment will be explored.

## Methods

### Multi-center Chinese-speaking RSF cohort in China

This is a cross-sectional study, with a total of 324 participants recruited from three memory clinics of hospitals in China (hereafter termed the RSF cohort: Ruijin Hospital Affiliated to Shanghai Jiao Tong University School of Medicine, Shanghai; Shanghai Sixth Hospital Affiliated to Shanghai Jiao Tong University School of Medicine, Shanghai; the First Hospital Affiliated to Zhejiang University, Zhejiang), in which 113 were NC (normal control), 95 were aMCI, and 116 participants were diagnosed with early phase AD. The registration number is ChiCTR2000036718 on the website associated with this study (https://www.chictr.org.cn). All participants (including the NC recruited among relatives of the aMCI and AD patients, with a request for NC participants also advertised) were recruited between August 2020 and July 2021 from the memory clinic of the RSF cohort centers mentioned above. The authors asserted that all procedures contributing to this work comply with the ethical standards of the relevant national and institutional committees on human experimentation and with the Helsinki Declaration of 1975, as revised in 2008. All procedures involving human subjects/patients were approved by the Ethics Committee of the RSF centers (approval number: 2020-261). All included individuals provided written consent.

### Clinical assessment in the RSF center

To exclude other causes of cognitive impairment, we performed cranial MRI or computed tomography (CT) to exclude confounding factors such as stroke or intracranial space-occupying lesions. Serum folic acid, vitamin B_12_ levels, and thyroid function were tested to exclude endocrine and metabolic disorders. Clinical and demographic data including age, gender, and level of education were also collected. All subjects underwent neuropsychological tests including the following: the Mini-Mental State Examination (MMSE), the Montreal Cognitive Assessment-Basic (MoCA-B), and Addenbrooke’s Cognitive Examination-III (ACE-III), scoring according to the Clinical Dementia Rating scale (CDR) and the Cookie-theft picture description task from the Boston Diagnostic Aphasia Scales [12–14].

After clinical assessment, the participants were categorized into three groups: (i) a NC group, who were considered as cognitively healthy after the clinical consultation; (ii) an AD group, whose diagnosis was based on the clinical probable criteria for diagnosis of AD issued by the National Institute on Aging-Alzheimer’s Association workgroups in 2011 [15]; and (iii) an aMCI group, in which patients had a memory complaint corroborated by at least one informant, and a diagnosis was conducted using the Petersen criteria [16]. Participants were excluded if they had any other neurological diseases, any systemic disease which can lead to cognitive dysfunction, psychiatric disorders, or severe hearing or vision impairment.

### English-speaking Cohort of the Pitt Center

The DementiaBank corpus, which is part of the TalkBank project, was used in the present study [17] and is an open-access database [4]. This corpus contained recordings of 104 controls and 208 dementia patients, from July 1983 to April 1988 (last modified in November 2018) involving the participants given a picture description task, which was originally designed for the Boston Diagnostic Aphasia Examination. The task required each participant to describe events depicted in the picture, the same as performed by participants in the China RSF center (Cookie Theft picture description task). We focus on the language character of aMCI individuals, in which most will convert to AD several years later. However, there were mainly mMCI (multi cognitive domain type) records and a lack of aMCI records in the DementiaBank. So, we decided to use individuals with mild AD with MMSE scores of over 24 to represent the early stage of AD, similar with the MMSE score range of aMCI individuals in the China multi-center cohort. There were 20 mild AD records after excluding unavailable records (recordings with a noisy background, speech time of over 60 s, or incomplete recordings), and 21 NC records were randomly selected form the control corpus. The diagnostic criteria for “Possible AD” or “Probable AD” determination were as specifically described in the study from Becker et al. [17]. In order to be consistent with the China RSF center and the previous study [18, 19], the samples with “Possible AD” and “Probable AD” labels are merged to compose the AD group in our study.

### Recording protocol and speech analysis

Subjects in the China RSF center performed a Cookie Theft picture description task, during which they were given a picture and were told to discuss everything they could see happening in the picture in 1 min while being recorded. The mean time duration of the records is 39.6 ± 17.7 s. The RSF cohort individuals’ speech was recorded under the following configuration parameters of Cool Edit Pro software: a frequency of 160000 Hz, creating a 16-bit mono recording, and environmental noise was limited to under 45 dB. The automatic speech recognition (ASR) software for cognitive impairment v1.3 (developed by our team, China Software Copyright number 2016SR164680) for speech analysis was used, according to our previous study [2]. The Pitt records (the mean time duration of the records is 39.0 ± 17.4 seconds) were converted to the audio configuration parameters identical to the RSF recording using the Cool Edit Pro software. Each sample was analyzed by ASR software for cognitive impairment using v1.3 to extract the speech/silence parameters. The sum of all silent periods divided by the total speech time is the definition of PSD (ratio of total silent pause duration to total speech duration), expressed as a percentage. The definition of basic parameters set in our software was according to Pakhomov et al. [20], who had developed the measurements of spontaneous speech from the Cookie Theft picture description task for patients with dementia. Silence is defined as the summed duration of all silent segments of the recording, including general short pauses, general long pauses, and hesitation-associated pauses.

### Task-based fMRI experiment

**Fig. 1:**
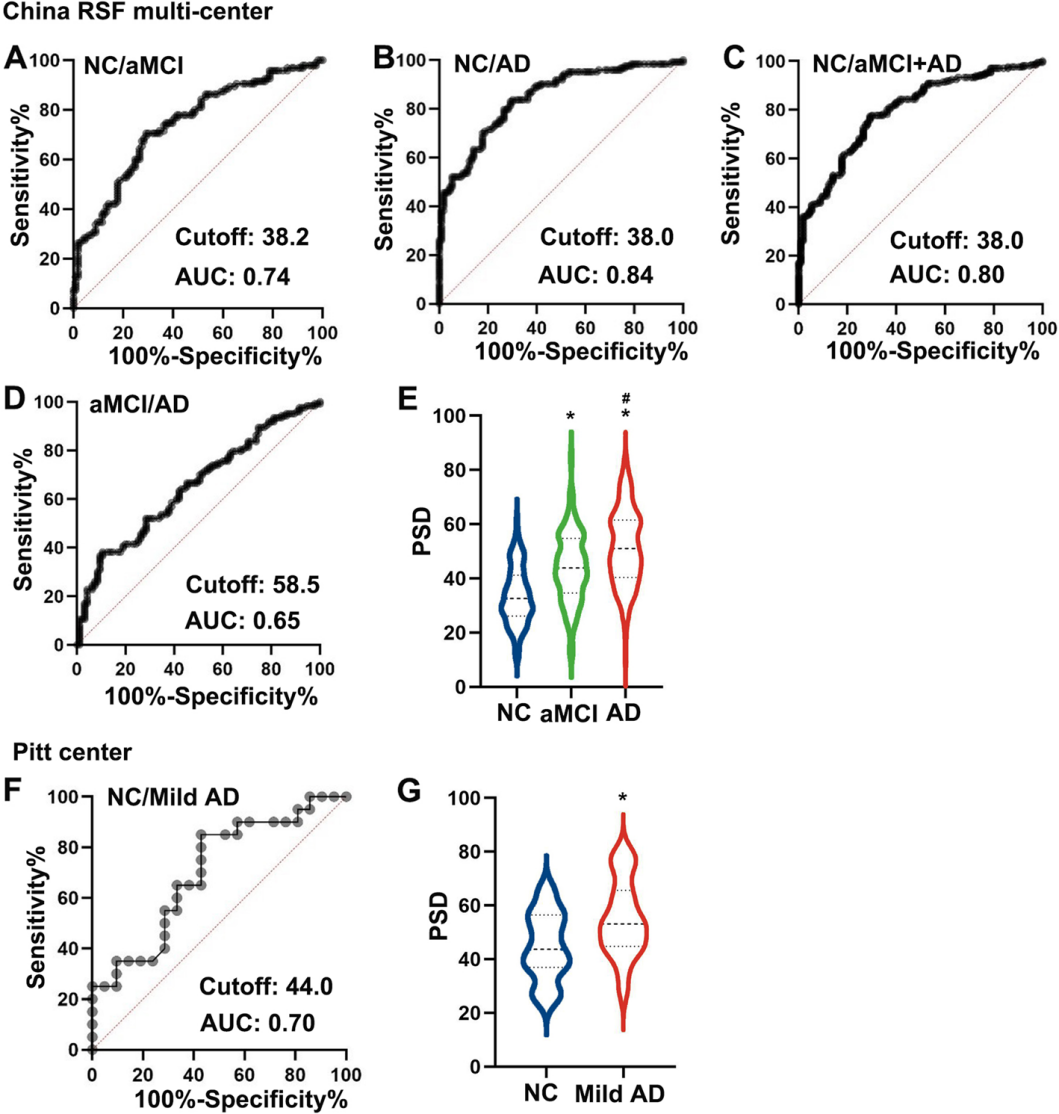
ROC curves and comparison of PSD among NC, MCI and AD patients in the China RSF multi-center (**A**–**E**) and Pitt center cohorts (**F**, **G**). The AUC and cutoff for PSD were 0.74 and 38.2 in NC/MCI (**A**), 0.84 and 38.0 in NC/aMCI (**B**), 0.80 and 38.0 in NC/aMCI+AD (**C**), and 0.65 and 58.5 in aMCI/AD (**D**). The comparison of PSD among NC, MCI, and AD (**E**, *P < 0.05 vs NC, ^#^*P* < 0.05 vs aMCI) in the RSF center and between NC and mild AD patients (**G**, *P < 0.05 vs NC) in the Pitt center cohort demonstrated an AUC of 0.70 with a PSD cutoff of 44.0 to distinguish NC from mild AD (**F**)

From current cohort in the Shanghai Ruijin center, a total of 48 right-handed individuals were recruited for further fMRI study. Seventeen participants were mild AD patients, fifteen were aMCI patients, and sixteen were NC. The inclusion and exclusion criteria were consistent with that of the current cohort as has been described above. In addition, patients with the following conditions were excluded: (a) moderate-to-severe AD indicated by MMSE < 15; (b) reading disability (or illiteracy); (c) abnormal findings in the brain MRI scan (e.g., tumors, stroke, hydrocephalus); (d) psychiatric disorders diagnosed by Diagnostic and Statistical Manual of Mental Disorders V (e.g., claustrophobia); and (e) refractive errors that cannot be corrected by MRI-supported eyeglasses. In addition to the neuropsychological scales assessed described above (MMSE, MoCA-B, ACE), MRI participants were further screened using the Boston naming test (BNT). Taking semantic and phonemic deficits into consideration, an fMRI verbal fluency task was adapted, as was shown in S Figure 1. The scanning protocol and processing methods are summarized in the supplementary materials.

### Statistical analysis

According our previous study [2], for continuous variables, normality and homogeneity of variance was tested. ANOVA (3 groups), or Student *T* test (2 groups) was used for normally distributed variables with equal population variance, and the non-parametric tests Kruskal-Wallis (3 groups) or Mann-Whitney *U* (2 groups) test was used for variables with nonhomogeneous variance. When the differences were statistically significant (*P <* 0.05) among three groups, post hoc multiple comparisons were further made; when the variance was equal, the Bonferroni method was used; otherwise, the Kruskal-Wallis test was used. Receiver operating characteristic (ROC) curves were plotted for PSD by calculating the sensitivity and specificity of their diagnostic power in NC, aMCI, and AD type dementia. To explore the correlation between the parameters, correlation analysis and stepwise multiple linear regression were used. All statistical analyses were performed using SPSS.

## Result

### The clinical characters of the subjects in China RSF center and Pitt center

There were 113 NC, 95 aMCI, and 116 AD patients in the China RSF multi-center cohort. Gender (female makeup in NC: 62.8%, aMCI: 56.8%, and AD: 52.8%) and educational level (NC: 12.2 ± 2.9 years, MCI: 11.5 ± 3.1 years, and AD: 11.3 ± 3.5 years) showed no significant difference among the NC, aMCI, and AD groups in the China RSF center cohort, and mean age was 67.6 ± 7.9, 73.0 ± 6.8, and 76.4 ± 8.2 years for the NC, MCI, and AD groups within this cohort (*P* < 0.001), respectively. However, there were significant differences between groups’ mean MMSE scores (NC: 28.7 ± 1.2, MCI: 26.2 ± 2.3, and AD: 19.0 ± 4.2), MoCA-B (NC: 26.0 ± 2.5, MCI: 20.7 ± 3.6, and AD: 14.5 ± 4.7), ACE-III performance (NC: 86.2 ± 7.4, MCI: 73.8 ± 9.3, and AD: 54.1 ± 13.6), and sub-items relating to fluency and language (Table 1, all *P* < 0.001), and the post-hoc comparison results are shown in S Table 1.

**Table 1:** Clinical characteristics of AD patients in the China RSF multi-center cohort

Multi-center in China	NC (*n* = 113)	MCI (*n* = 95)	AD (*n* = 116)	Statistics	*P* value
Gender, female, *n*, %	71 (62.8%)	54 (56.8%)	61 (52.6%)	2.617	0.270
Age (years)	67.6 ± 7.9	73.0 ± 6.8	76.4 ± 8.2	39.104	0.000
Education (years)	12.2 ± 2.9	11.5 ± 3.1	11.3 ± 3.5	5.293	0.071
ApoE e4 type^a^, *n*, %	11/54, 20.4%	20/57, 35.1%	39/68, 57.4%	17.754	0.000
MMSE	28.7 ± 1.2	26.2 ± 2.3	19.0 ± 4.2	234.738	0.000
MoCA-B	26.0 ± 2.5	20.7 ± 3.6	14.5 ± 4.7	212.456	0.000
ACE	86.2 ± 7.4	73.8 ± 9.3	54.1 ± 13.6	200.906	0.000
ACE-fluence	9.3 ± 2.0	7.6 ± 2.0	5.2 ± 2.2	125.069	0.000
ACE-language	23.7 ± 2.5	20.3 ± 3.6	16.6 ± 5.2	120.305	0.000
PSD	33.5 ± 10.5	44.1 ± 12.8	51.2 ± 14.3	86.866	0.000

^a^Data on ApoE e4 type was available in 54, 57, and 68 of NC, aMCI, and AD patients, respectively. For acronyms, please refer to the “Methods” section

Regarding the Pitt center, 20 mild AD patients (MMSE ≥ 24) and 21 NC individuals randomly selected had clinical characteristics shown in S Table 2. PSD was also significantly different between NC and mild AD patients.

### PSD as a biomarker for aMCI and AD

**Fig. 2:**
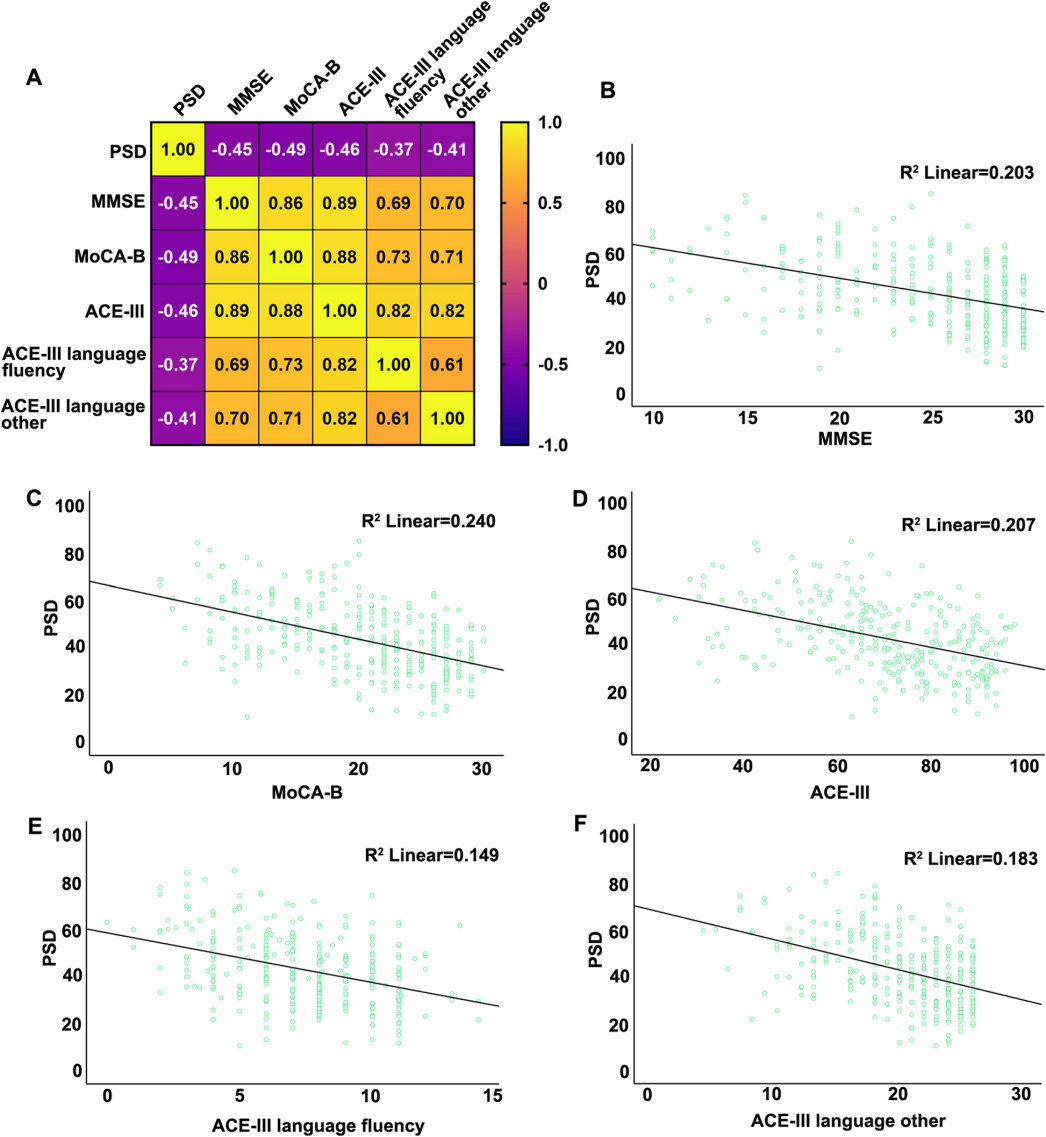
The correlation analysis of PSD with cognitive performance. The heatmap (**A**) and the scatter dot of PSD with MMSE (**B**), MoCA (**C**), ACE-III (**D**), ACE-language fluency (**E**), and ACE-language-other (**F**)

In the China RSF center cohort, compared with NC subjects, aMCI and AD patients had significantly increased PSD (Table 1, Fig. 1, *P* < 0.001), and PSD inversely correlated with cognitive performance (Fig. 2, S Table 3, *P* < 0.001). Following linear regression analysis, the variables representing aMCI and AD status of individuals in the cohort were significantly correlated with PSD after adjusting for age (S Table 4). The ROC curves comparing PSD-based classification sensitivity and specificity among NC, aMCI, and AD patients are shown in Fig. 1A–D. The AUCs of the curves are 0.74, 0.84, 0.80, and 0.65 in NC/aMCI, NC/AD, NC/aMCI+AD, and aMCI/AD, and the sensitivity and specificity of NC/aMCI, NC/AD, NC/aMCI+AD, and aMCI/AD is 0.71/0.71, 0.84/0.70, 0.78/0.79, and 0.85/0.43 respectively. The optimal cutoff for PSD in NC/aMCI, NC/AD, and NC/aMCI+AD was around 38.0 for each classifier using the SPSS ROC package. The distribution and comparison of PSD in NC, aMCI, and AD groups is presented in Fig. 1E. In the Pitt center cohort, PSD was verified as a biomarker to differentiate mild AD patients from NC (AUC of NC/mild AD is 0.70, Fig. 1F), and the difference in mean PSD between NC and mild AD patients was significant (Fig. 1G, *P* = 0.018).

### Verbal fluency-based fMRI network

Demographic, neuropsychological, and language characteristics of fMRI participants are shown in S Table 5. There was no difference in age, gender distribution, nor education level among the NC, aMCI, and AD groups (*P*>0.05). The results of neuropsychological assessments and PSD for the fMRI participants was consistent with that of the RSF Center cohort as well.

**Fig. 3:**
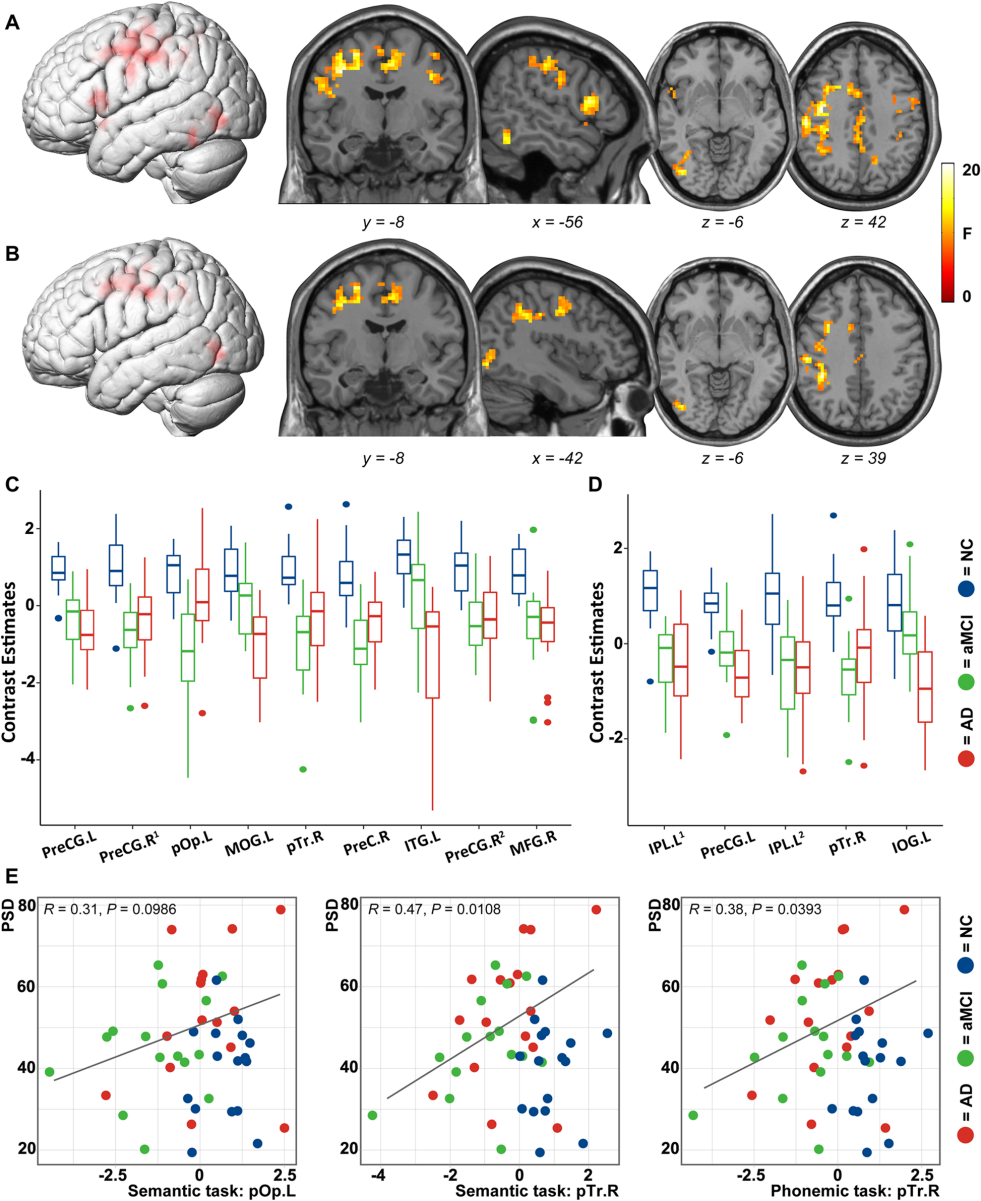
Group differences in fluency tasks. Clusters that activated at different levels among NC, aMCI, and AD in the semantic task (**A** and **C**) and the phonemic task (**B** and **D**) are presented respectively (one-way ANOVA, voxel-level *P* < 0.001, cluster-level *P* < 0.05, FWE corrected). Coordinates of clusters are listed in S Table 6. Results of partial correlation analysis between PSD and BOLD signals of the clusters in patient group (AD+aMCI) were presented in **E** and S Table 7 (age and gender controlled). L, left; R, right; PreCG, precentral gyrus; pOp, pars opercularis; pTr, pars triangularis; MOG, middle occipital gyrus; PreC, precuneus; ITG, inferior temporal gyrus; MFG, middle frontal gyrus; IPL, inferior parietal lobule; IOG, inferior occipital gyrus. Superscript digits one and two (^1, 2^) indicates the following: clusters in the same anatomical region

Clusters showing significant difference in ANOVA analysis are presented in Fig. 3 and S Table 6 (cluster-level *P* < 0.05, FWE corrected). In the semantic task (Fig. 3A), the peak foci were mainly located at the bilateral precentral gyrus (PreCG), left pars opercularis (pOp) and pars triangularis (pTr), left middle occipital gyrus (MOG), and right precuneus and pTr. There was a significant positive correlation between the BOLD signals of all clusters except the left pOp and semantic fluency sub-scores of ACE-III (S Table 7). In the phonemic task (Fig. 3B), areas activated differently across groups were confined to the left PreCG, inferior parietal lobule (IPL), inferior occipital gyrus (IOG) and right pTr. Among them, the left IPL, PreCG, and right pTr were found to be associated with phonemic fluency sub-scores of ACE-III (S Table 7). In the post hoc analysis (Fig. 3C, D; S Table 8), we observed that in both AD and aMCI nearly all clusters showed remarkable deactivation in comparison with the NC group (*P* < 0.05, Bonferroni corrected). Compared with aMCI, the response of AD patients to fluency tasks in most of these clusters declined further except left pOp (*P* < 0.05, Bonferroni corrected), right pTr and PreCG (not significant) activated at a relatively higher level. The results remained robust basically when the age, gender, and education level of subjects were regressed out as nuisance covariates. In addition, in the semantic fluency–fixation contrasts, a cluster in the left cerebellum crus I was observed to deactivate in AD/aMCI (S Fig. 2). No group differences were detected in other contrasts, i.e., repetition > fixation; phonemic fluency > fixation; semantic/phonemic fluency > repetition; semantic fluency > phonemic fluency.

Unexpectedly, in the semantic task, the higher activation level of the right pTr was associated with the higher PSD in AD and aMCI (*R* = 0.43, *P* = 0.0148). While controlling for the effect of age and gender, we found the correlation between the right pTr and PSD was significant in both semantic and phonemic tasks (Fig. 3E, S Table 7). No significant correlation was observed in other clusters with PSD performance.

## Discussion

In the present study, we performed a comprehensive analysis of language components in NC, aMCI, and AD individuals, including characteristic PSD in both the China RSF multi-center cohort and the DementiaBank corpus of the Pitt center, with a task-based fMRI study of the underlying functional neural substrates. Our results show that PSD was both sensitive and specific in the diagnosis of aMCI and AD. Meanwhile, as another side of speech pause (PSD), verbal fluency was involved with functional alteration in the language network covering the bilateral PreCG, left ITG, and IPL, together with Broca’s area and its counterpart in the right hemisphere.

Language impairment is a core feature of AD [21]. Prior studies have shown a link between AD symptom severity and declining speech and language capability [3]. The data based on speech analysis of AD patients indicated that combined language characteristics provided a diagnostic accuracy of over 80% [5]. The first study using automatic speech analysis to identify MCI and AD patients compared the voices of healthy older adults and patients with extracted features that showed significant differences in several tasks and obtained the best combination through machine-learning methods, with an accuracy of 79% [22]. Another study used the features related to duration, speech rate, articulation rate, and pauses to obtain a 78.8% accuracy for MCI [23]. Our previous study on the combined language characteristics of PSD showed this metric better discriminated aMCI form NC with a limited sample size [2]. However, there was a lack of a language-specific parameter within that sample from multiple centers which could transgress language-specific differences. Therefore, in the present study, results from a considerably larger sample of NC, aMCI, and AD subjects in a multi-center cohort and an additional English-speaking validation cohort further confirmed that PSD as a single parameter is a sensitive indicator of aMCI and AD, both discriminated via an optimal PSD cutoff that achieves 80% accuracy (AUC of 0.8). The ability of PSD to discriminate mild AD was also validated in the Pitt center cohort. These results indicate that PSD is a non-invasive and easily accessed reliable biomarker for diagnosis of early-stage AD and is not restricted to different types of language or dialect, in both Chinese- and English-speaking populations. Although there is significant difference of PSD between aMCI and AD, the poor AUC (aMCI/AD, 0.65) and specificity (aMCI/AD, 0.45) indicated that PSD could not well predict aMCI due to AD.

Compared with task-based tests of episodic memory and other cognitive domains, a language task proved to be more sensitive and accurate in early identification of AD by fMRI [24]. To unveil the mechanism underlying the increase in PSD in AD/aMCI patients, we conducted a block-design fMRI paradigm focusing on verbal fluency, because pauses as a potential key factor correspond to speech fluency, which is mainly composed by semantic and phonemic fluency [8]. As it should be, the fMRI differences could still be ascribed to functional alterations in multiple cognitive abilities; thus, we put emphasis on discussing the clusters in canonical areas supporting language processing in young adults or older adults [25–27]. Echoing the aforementioned studies [28–30], for patients with AD or aMCI, there were fewer brain areas recruited in the semantic-lexical processing, in comparison with normal aging. One unanticipated finding was that this type of deactivation had emerged at as early as aMCI phase, while the corresponding symptoms did not become evident until the dementia phase [31]. On the contrary, the increased recruitment of brain resources in response to semantic tasks was found in older NC with or without a high risk of AD [32, 33], suggesting that physiological compensation in aging may have disappeared at the early phase of AD. In the semantic fluency task, clusters in left pTr, ITG, and PreCG (the lower part at the junction with pOp, precisely), as components of semantic network [26], deactivated markedly in AD/aMCI patients, partly explaining why semantic processing is disrupted [24]. Particularly, despite the decreased activation of the left cerebellum associated with declining fluency scores, the role it plays in language processing remains unknown [34]. Another interesting finding was that compared with the aMCI group, Broca’s area and its homologous areas in the right hemisphere in AD patients activated at a relatively higher level. Moreover, in AD and aMCI, the activation level of the right pTr was positively related to PSD, suggesting it may have a crucial role in pause-related network. Different from the role the left pTr play in the language function, the right pTr is considered to be a hub region supporting social cognition and control network [35]. Plenty of studies have found that there could be extra inter-hemisphere recruitment for the fluency task and a latent recovery of language function after brain damage [36–38]. However, contrary to what is seen in normal aging [33, 39], the reduced lateralization in pTr/pOp of AD patients did not result in enhanced fluency performance nor decreased speech pause, which could be better interpreted as a failed attempt or “decompensation” of the language network.

No group differences were detected in other contrasts, including repetition vs. fixation, semantic/phonemic fluency vs. repetition, and semantic vs. phonemic fluency. We supposed this could result from the fact that in older adults a more widely-distributed language network has been recruited even in the resting-state [40, 41], making the response to fixation, low- and high-difficulty tasks look almost the same.

## Strengths and limitations

Firstly, this is a multi-center study identifying PSD differences in early stages of AD and its associated brain structures, combined with verification of the PSD effect in early AD in the Pitt center cohort; the underling mechanism of verbal fluency due to changes in specific brain areas was explored with task-based fMRI. However, our investigation is a cross-sectional study without observation of longitudinal changes in the patients. Secondly, there was limited enrollment of fMRI participants in a single center and a lack of electric voice-monitoring devices installed with the fMRI stimulus-presenting system, which could show how participants performed in the scanner. Lastly, for the various types of pauses distributed throughout speech recordings, more fine-grained analyses could be considered in detail in future studies.

## Conclusion and hypothesis

This study provided new evidence that PSD is sensitive for diagnosis of early-stage AD or aMCI. At as early as aMCI phase, the brain response to fluency tasks was inhibited markedly, partly explaining why PSD was elevated simultaneously.

## Supplementary Information

**Additional file 1: Supplemental Figure 1.** fMRI task paradigm. **Supplemental Figure 2.** Group difference in the Semantic Fluency-Fixation contrasts and its behavioral significance. One-way ANOVA, cluster-level *P* < 0.05, FWE corrected. **Supplemental Table 1.** The Post-hoc comparisons of the clinical characteristics in the China RSF multi-center cohort (Bonferroni corrected). **Supplemental Table 2.** Clinical characteristics of NC and AD patients in the Pitt center cohort. **Supplemental Table 3.** Correlation between PSD and cognition. **Supplemental Table 4.** The linear regression analysis of the variables correlated with PSD after adjusting for age. **Supplemental Table 5.** Demographic and neuropsychological/language characteristics of fMRI participants. **Supplemental Table 6.** Significant clusters in three linguistic tasks showing different activation by ANOVA analysis. **Supplemental Table 7.** Results of correlation analysis. # The spearman correlation coefficients were calculated between the average BOLD signal and the relevant fluency score of ACE-III. * The partial correlation coefficients were calculated between the average BOLD signal and PSD in the patient group. **Supplemental Table 8.** Post hoc analysis of cluster activation. Bold indicated significance (Bonferroni corrected).

## Data Availability

The datasets used and/or analyzed during the current study are available from the corresponding author on reasonable request.
