# Supplementary material for: Speech silence character as a diagnostic biomarker of early cognitive decline and its functional mechanism: a multicenter cross-sectional cohort study
Source: BMC Med. 2022 Nov 7;20:380. doi: 10.1186/s12916-022-02584-x (PMC9639269; doi:10.1186/s12916-022-02584-x)
Supplement: Supplementary file 1 — Additional file 1: Supplemental Figure 1. fMRI task paradigm. Supplemental Figure 2. Group difference in the Semantic Fluency-Fixation contrasts and its behavioral significance. One-way ANOVA, cluster-level P < 0.05, FWE corrected. Supplemental Table 1. The Post-hoc comparisons of the clinical characteristics in the China RSF multi-center cohort (Bonferroni corrected). Supplemental Table 2. Clinical characteristics of NC and AD patients in the Pitt center cohort. Supplemental Table 3. Correlation between PSD and cognition. Supplemental Table 4. The linear regression analysis of the variables correlated with PSD after adjusting for age. Supplemental Table 5. Demographic and neuropsychological/language characteristics of fMRI participants. Supplemental Table 6. Significant clusters in three linguistic tasks showing different activation by ANOVA analysis. Supplemental Table 7. Results of correlation analysis. # The spearman correlation coefficients were calculated between the average BOLD signal and the relevant fluency score of ACE-III. * The partial correlation coefficients were calculated between the average BOLD signal and PSD in the patient group. Supplemental Table 8. Post hoc analysis of cluster activation. Bold indicated significance (Bonferroni corrected). [file 12916_2022_2584_MOESM1_ESM.docx]

**Methods**

**fMRI scanning**

All scans were performed on a 1.5T Siemens scanner (MAGNETOM Aera) with a 20-channel head coil at Ruijin Hospital, Shanghai. High-resolution structural scans were acquired using 3D T1-weighted magnetization prepared rapid acquisition gradient echo (3D T1-MPRAGE) sequence, with the following parameters: repetition time (TR) = 2400ms, echo time (TE) = 3.81ms, inversion time (TI) = 1000ms, flip angle = 8°, field of view (FOV) = 240 × 240mm, matrix 192 × 192, 160 sagittal slices, slice thickness = 1.20 mm. Task fMRI data were acquired using a gradient echo T2-weighted pulse sequence (BOLD) with the following parameters: TR = 2000ms, TE = 30ms, flip angle = 90°, FOV = 192mm × 192mm, matrix = 64 × 64, 31 axial slices, slice thickness = 3.5mm, and a total scanning time of 8 minutes. To confine head movement, participants were instructed to supine quietly with heads immobilized by cushion supports during the scan.

**fMRI task paradigm**

Taking semantic and phonemic deficits into consideration, a fMRI verbal fluency task was adapted according to previous studies^1-4^. As was shown in **S Figure 1**, the task was composed of six blocks, each of which had one resting period and three language-related activation periods. During each block, participants were first instructed to lie passively with eyes focusing on a cross in the middle of the screen for 20s. Then participants were asked to engage in a repetitive task (repeat a single word, ‘Pencil,’ continuously), a semantic task (speak nouns of one category, e.g. animals), and a phonemic task (speak words that start with one Chinese character, e.g. ‘Da’ (referred to as ‘big’ in English)) in a pseudo-randomized order, with each task lasting for 20s. A total of 6 common categories and 6 frequently-used characters were chosen on the basis of matching difficulty. Both as the reference tasks, the cross-fixation and repetition task were both designed to serve as controls for targeting specific components of language processing^5,6^. All stimuli were presented in Chinese and projected to a mirror fixed to the head coil from an LCD screen in the scanning room. To ensure the process proceeded smoothly inside the scanner, all patients and controls were well-informed of the experimental protocol before the scan and instructions for the whole task were shown in full-screen during T1-weighed image scanning. Besides, the stimuli-presenting device allowed us to record the number of words simultaneously by instructing subjects to press the button every time they enumerated a word in the fluency tasks.

**fMRI data processing and statistical analyses**

A specialized radiologist excluded one AD patient because of excessive artifacts. fMRI data were preprocessed using the toolbox DPARSF V6.0^7^ equipped on MATLAB R2013b. For each participant, time-slice correction was applied on functional images, adjusting for temporal differences between interleaved sections. Realignment was adopted to minimize the effect of head movement, during which 12 motion parameters were obtained for further regression in first-level analysis. Then, functional images were spatially normalized to Montreal Neurological Institute (MNI) space using T1 unified segmentation and were checked cautiously to what extent functional images matched MNI templates. Last, smoothing with a 4×4×4 mm^3^ full width at half-maximum Gaussian Kernel was applied to the normalized images.

Statistical analysis of the image data was performed using SPM12 (https://[www.fil.ion.ucl.ac.uk/spm/](http://www.fil.ion.ucl.ac.uk/spm/)). For each subject, a general linear model was established respectively by specifying the onsets and durations (20s) of each language task for the six blocks. The general linear model contrasts included: fixation/repetition/fluency; repetition > fixation; semantic/phonemic fluency > fixation; semantic/phonemic fluency > repetition; semantic fluency > phonemic fluency. To find an activation difference between groups, analysis of variance (ANOVA) was conducted in a comparison model using the contrast images. A voxel level threshold of *P*<0.001 was set for group analysis and only clusters surviving multiple correction of family wise error (FWE) rate <0.05 were preserved. Then, the average activation value of each cluster was extracted using the marsbar toolbox (https://marsbar.sourceforge.net/) and input into further correlation analysis with behavioral scores using ggm package from R version 4.1.0 (https://www.r-project.org/). Specially, in the patient group (AD + aMCI), partial correlation was used to find the correlation between the activation level and PSD with the effect of age and gender being controlled. The number of words were not analyzed because we found it too hard for patients with amnestic mild impairment (aMCI) or Alzheimer’s disease (AD) to multitask during the scanning process.

**References:**

1. Pereira JB, Junque C, Bartres-Faz D, et al. Modulation of verbal fluency networks by transcranial direct current stimulation (tDCS) in Parkinson's disease. *Brain Stimul* 2013; **6**(1): 16-24.

2. Birn RM, Kenworthy L, Case L, et al. Neural systems supporting lexical search guided by letter and semantic category cues: a self-paced overt response fMRI study of verbal fluency. *NEUROIMAGE* 2010; **49**(1): 1099-107.

3. Marsolais Y, Perlbarg V, Benali H, Joanette Y. Age-related changes in functional network connectivity associated with high levels of verbal fluency performance. *Cortex; a journal devoted to the study of the nervous system and behavior* 2014; **58**: 123-38.

4. Metzger FG, Schopp B, Haeussinger FB, et al. Brain activation in frontotemporal and Alzheimer's dementia: a functional near-infrared spectroscopy study. *Alzheimers Res Ther* 2016; **8**(1): 56.

5. Vigneau M, Beaucousin V, Herve PY, et al. Meta-analyzing left hemisphere language areas: phonology, semantics, and sentence processing. *NEUROIMAGE* 2006; **30**(4): 1414-32.

6. Crosson B, McGregor K, Gopinath KS, et al. Functional MRI of language in aphasia: a review of the literature and the methodological challenges. *Neuropsychol Rev* 2007; **17**(2): 157-77.

7. Yan CG, Wang XD, Zuo XN, Zang YF. DPABI: Data Processing & Analysis for (Resting-State) Brain Imaging. *Neuroinformatics* 2016; **14**(3): 339-51.

8. Mohanty R, Gonzalez-Burgos L, Diaz-Flores L, et al. Functional Connectivity and Compensation of Phonemic Fluency in Aging. *Frontiers in Aging Neuroscience* 2021; **13**


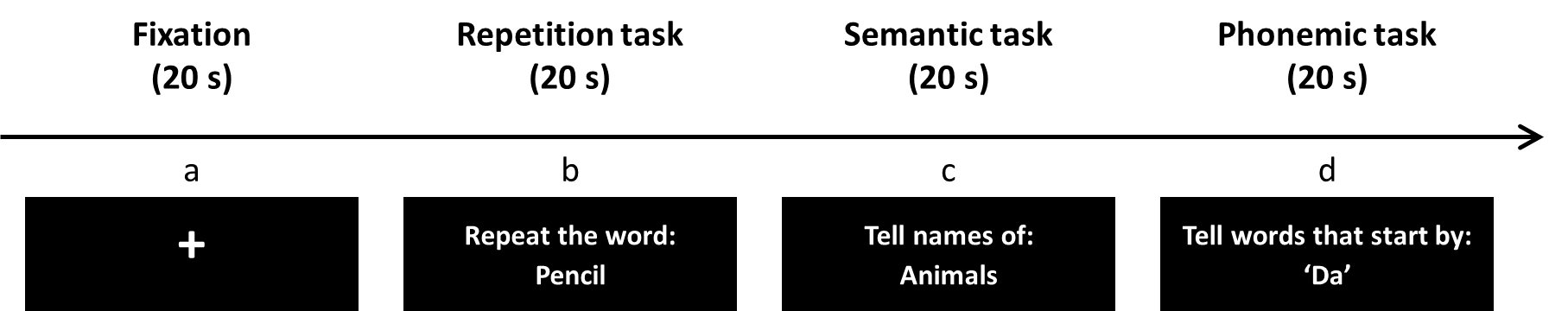
**Supplemental figure 1. fMRI task paradigm.** The fMRI verbal fluency task was composed of 6 blocks, each of which had 4 parts: a) fixation; b) a repetition task (repeating a single word, ‘Pencil,’ continuously); c) a semantic task (speaking names of one category, e.g., animals); d) a phonemic task (speak words that start with one Chinese character, e.g., ‘Da’, or ‘Xiao’). Performance of each block was randomized by e-prime 2.0 and lasted 80s in total (20s per task).

**Supplemental figure 2. Group difference in the Semantic Fluency-Fixation contrasts and its behavioral significance. One-way ANOVA, cluster-level *P* < 0.05, FWE corrected.**


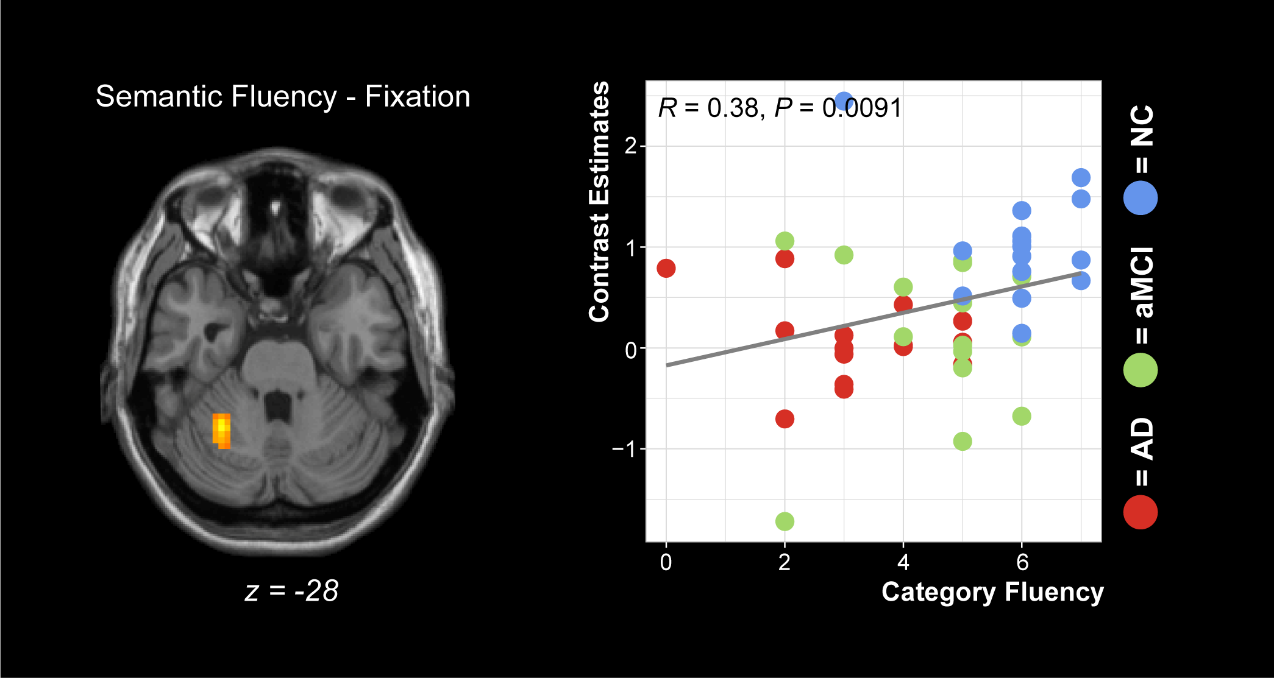


**Supplemental table 1.** The Post-hoc comparisons of the clinical characteristics in the China RSF multi-center cohort (Bonferroni corrected).

| group *vs* group | | | Statistics | *P-*value |
| --- | --- | --- | --- | --- |
| age | NC | aMCI | -60.350 | 0.000 |
|  | NC | AD | -73.126 | 0.000 |
|  | aMCI | AD | -12.776 | 0.324 |
| MMSE | NC | aMCI | 109.593 | 0.000 |
|  | NC | AD | 187.929 | 0.000 |
|  | aMCI | AD | 78.336 | 0.000 |
| MoCA-B | NC | aMCI | 87.321 | 0.000 |
|  | NC | AD | 175.492 | 0.000 |
|  | aMCI | AD | 88.171 | 0.000 |
| ACE | NC | aMCI | 91.045 | 0.000 |
|  | NC | AD | 165.948 | 0.000 |
|  | aMCI | AD | 74.903 | 0.000 |
| ACE fluency | NC | aMCI | 72.482 | 0.000 |
|  | NC | AD | 130.575 | 0.000 |
|  | aMCI | AD | 57.734 | 0.000 |
| ACE language | NC | aMCI | 51.686 | 0.000 |
|  | NC | AD | 128.055 | 0.000 |
|  | aMCI | AD | 76.369 | 0.000 |
| PSD | NC | aMCI | -72.794 | 0.000 |
|  | NC | AD | -114.261 | 0.000 |
|  | aMCI | AD | -41.467 | 0.000 |

Abbreviations: AD: Alzheimer’s disease; aMCI: amnestic mild cognitive impairment; NC: normal controls; MMSE: Mini-mental State Examination; MoCA-B: Montreal Cognitive Assessment-Basic; ACE: Addenbrooke’s Cognitive Examination-III; PSD: percentage of silence duration.

**Supplemental table 2.** Clinical characteristics of NC and AD patients in the Pitt center cohort.

| Pitt center | NC (n=21) | mild AD (n=20) | Statistics | *P* Value |
| --- | --- | --- | --- | --- |
| Gender, Female, n | 11 (52.3%) | 11 (55%) | 204.500 | 0.868 |
| Age (years) | 64.8 ± 7.6 | 71.9 ± 9.0 | -2.757 | 0.009* |
| Education (years) | 14.1 ± 2.4 | 11.8 ± 3.2 | 116.500 | 0.014* |
| MMSE | 29.0 ± 1.1 | 25.1 ± 1.6 | 22.000 | 0.000* |
| PSD | 44.7 ± 12.5 | 55.0 ± 14.3 | -2.475 | 0.018* |

* Significant at *P* < 0.05 *vs* NC

**Supplemental table 3** Correlation between PSD and cognition.

|  |  | MMSE | MoCA-B | ACE-III | ACE-language fluency | ACE-language-other |
| --- | --- | --- | --- | --- | --- | --- |
| PSD | Correlation coefficient | -.446^**^ | -.493^**^ | -.462^**^ | -.367^**^ | -.407^**^ |
|  | Sig. （2-tailed） | 0.000 | 0.000 | 0.000 | 0.000 | 0.000 |
|  | | | | | | |

** Correlation is significant at the 0.001 level (2-tailed)

**Supplemental table 4.** The linear regression analysis of the variables correlated with PSD after adjusting for age

| Model | | Unstandardized Coefficients | | Standardized Coefficients | t | Sig. | 95.0% Confidence Interval for B | |
| --- | --- | --- | --- | --- | --- | --- | --- | --- |
|  |  | B | Std. Error | Beta |  |  | Lower Bound | Upper Bound |
|  | (Constant) | 26.130 | 6.499 |  | 4.020 | .000 | 13.339 | 38.920 |
|  | MCI group | 10.001 | 1.793 | .330 | 5.578 | .000 | 6.473 | 13.530 |
|  | AD group | 15.465 | 1.868 | .508 | 8.280 | .000 | 11.790 | 19.141 |
|  | Age | .109 | .095 | .063 | 1.147 | .252 | -.078 | .295 |
|  | | | | | | | | |

**Supplemental table 5.** Demographic and neuropsychological/language characteristics of fMRI participants.

|  | | | | |
| --- | --- | --- | --- | --- |
|  | AD (n=16) | aMCI (n=15) | NC (n=16) | *P*-value |
| Age (in years) | 75.81 ± 8.22 | 77.73 ± 6.23 | 72.94±8.68 | 0.238 |
| Gender (M:F) | 8:8 | 7:8 | 6:10 | 0.767 |
| Disease Duration (in years) | 4.94 ± 2.72 | 2.65 ± 1.89 | / | 0.011 |
| Education (in years) | 11.94 ± 4.28 | 13.20 ± 2.88 | 13.38 ± 2.16 | 0.401 |
| MMSE | 20.25 ± 3.79 | 26.53 ± 2.26 | 29.12 ± 0.96 | <0.001 ^a, b, c^ |
| MoCA-B | 16.38 ± 4.30 | 20.60 ± 2.95 | 26.75 ± 2.29 | <0.001 ^a, b, c^ |
| ACE-III | 63.56 ± 11.42 | 83.20 ± 5.65 | 93.06 ± 3.17 | <0.001 ^a, b, c^ |
| Attention | 13.25 ± 2.46 | 16.07 ± 1.44 | 17.88 ± 0.34 | <0.001 ^a, b, c^ |
| Memory | 12.19 ± 4.23 | 21.47 ± 3.07 | 24.38 ± 1.71 | <0.001 ^a, b, c^ |
| Fluency | 6.94 ± 1.84 | 8.60 ± 1.76 | 10.88 ± 2.06 | <0.001 ^b, c^ |
| Phonetic fluency | 3.62 ± 1.02 | 4.07 ± 0.80 | 4.94 ± 1.24 | 0.009 ^b^ |
| Category fluency | 3.31 ± 1.40 | 4.53 ± 1.30 | 5.94 ± 1.00 | <0.001 ^b, c^ |
| Language | 19.06 ± 4.31 | 21.73 ± 2.81 | 24.31 ± 1.40 | <0.001 ^b^ |
| Repetition | 2.75 ± 1.06 | 3.4 ± 0.91 | 3.69 ± 0.6 | 0.018 ^b^ |
| Visuospatial | 12.12 ± 2.78 | 15.33 ± 1.23 | 15.62 ± 0.62 | <0.001 ^a, b^ |
| BNT | 18.94 ± 4.75 | 22.00 ± 4.09 | 25.69 ± 2.52 | <0.001 ^b, c^ |
| PSD | 53.12 ± 16.31 | 45.39 ± 12.63 | 39.83 ± 11.63 | 0.030 ^b^ |

Measures are reported as mean ± standard deviation. M, male; F, female. *P*-value were obtained using ANOVA or Kruskal-Wallis test where appropriate. ^a^ Significant difference was found in comparison between AD and aMCI; ^b^ Significant difference was found in comparison between AD and NC; ^c^ Significant difference was found in comparison between aMCI and NC (*P*<0.05, Bonferroni corrected).

**Supplemental table 6.** Significant clusters in three linguistic tasks showing different activation by ANOVA analysis.

| Brain area (Brodmann’s area, BA) | Cluster size | Cluster-level  *P*-value (corrected) | Z value at  local maximum | MNI coordinates | | | | | |
| --- | --- | --- | --- | --- | --- | --- | --- | --- | --- |
|  |  |  |  | *x* |  | *y* |  | *z* |  |
| Repetition task |  |  |  |  |  |  |  |  |  |
| R Superior Temporal Gyrus (BA 48) | 34 | 0.029 | 4.56 | 57 |  | -36 |  | 24 |  |
| R Supramarginal Gyrus (BA 40) |  |  | 3.73 | 63 |  | -42 |  | 33 |  |
| L Inferior Parietal Gyrus (BA 40) | 55 | 0.003 | 4.30 | -39 |  | -39 |  | 39 |  |
| L Angular Gyrus (BA 40) |  |  | 3.97 | -39 |  | -54 |  | 36 |  |
| L Precentral Gyrus (BA 6) | 62 | 0.001 | 4.18 | -36 |  | -9 |  | 45 |  |
| L Inferior Occipital Gyrus (BA 19) | 31 | 0.042 | 4.10 | -39 |  | -75 |  | -3 |  |
| L Middle Cingulate (BA 24) | 38 | 0.018 | 4.09 | -3 |  | 9 |  | 42 |  |
| L Supplementary Motor Area (BA 32) |  |  | 3.82 | -9 |  | 18 |  | 45 |  |
| R Middle Cingulate | 52 | 0.004 | 4.05 | 6 |  | -12 |  | 48 |  |
| L Middle Cingulate (BA 23) |  |  | 3.85 | -3 |  | -21 |  | 42 |  |
| L Precuneus | 32 | 0.037 | 3.84 | -3 |  | -48 |  | 48 |  |
| R Precentral Gyrus (BA 6) | 45 | 0.008 | 3.74 | 42 |  | -15 |  | 51 |  |
| Semantic task |  |  |  |  |  |  |  |  |  |
| L Precentral Gyrus (BA 6) | 1058 | 0.000 | 5.01 | -39 |  | -6 |  | 42 |  |
| L Inferior Parietal Gyrus (BA 3) |  |  | 4.92 | -54 |  | -21 |  | 42 |  |
| R Precentral Gyrus (BA 6) | 109 | 0.000 | 4.63 | 24 |  | -24 |  | 57 |  |
| L Pars Opercularis (BA 44) | 59 | 0.003 | 4.48 | -57 |  | 15 |  | 12 |  |
| L Pars Triangularis (BA 45) |  |  | 3.56 | -48 |  | 24 |  | 9 |  |
| L Middle Occipital Gyrus (BA 37) | 88 | 0.000 | 4.42 | -48 |  | -66 |  | 0 |  |
| L Inferior Occipital Gyrus (BA 19) |  |  | 4.41 | -42 |  | -75 |  | -6 |  |
| R Pars Triangularis (BA 45) | 40 | 0.020 | 4.29 | 57 |  | 27 |  | 18 |  |
| R Precuneus | 36 | 0.031 | 4.19 | 12 |  | -57 |  | 45 |  |
| L Inferior Temporal Gyrus (BA 37) | 32 | 0.049 | 4.18 | -57 |  | -54 |  | -18 |  |
| R Precentral Gyrus (BA 6) | 40 | 0.020 | 4.17 | 39 |  | -9 |  | 39 |  |
| R Middle Frontal Gyrus (BA 6) | 86 | 0.000 | 4.07 | 33 |  | 6 |  | 48 |  |
| Phonemic task |  |  |  |  |  |  |  |  |  |
| L Inferior Parietal lobule (BA 40,3)^1^ | 90 | 0.000 | 4.85 | -39 |  | -39 |  | 39 |  |
| L Precentral Gyrus (BA 6) | 441 | 0.000 | 4.67 | -33 |  | 9 |  | 45 |  |
| L Inferior Parietal lobule (BA 3,2)^2^ | 43 | 0.012 | 4.67 | -54 |  | -21 |  | 39 |  |
| R Pars Triangularis (BA 45,44) | 37 | 0.024 | 4.45 | 57 |  | 27 |  | 18 |  |
| L Inferior Occipital Gyrus (BA 19) | 60 | 0.002 | 4.21 | -42 |  | -75 |  | -6 |  |
| L Middle Occipital Gyrus (BA 37) |  |  | 4.16 | -48 |  | -66 |  | 0 |  |
| Semantic fluency > Fixation |  |  |  |  |  |  |  |  |  |
| L Cerebellum Crus Ⅰ | 65 | 0.037 | 4.2 | -39 |  | -60 |  | -36 |  |

Abbreviations: L, left; R, right; BA, Brodmann's area.

**Supplemental table 7. Results of correlation analysis.** ^#^ The spearman correlation coefficients were calculated between the average BOLD signal and the relevant fluency score of ACE-Ⅲ. ^*^ The partial correlation coefficients were calculated between the average BOLD signal and PSD in the patient group.

| **Brain area (peak)** | | | **ACE**-**Ⅲ fluency^#^** | | | |  | **PSD^*^** | |
| --- | --- | --- | --- | --- | --- | --- | --- | --- | --- |
|  |  |  | **R** (all) | ***P*** | **R** (AD+aMCI) | ***P*** |  | **R** (AD+aMCI) | ***P*** |
| **Semantic task** | | | | | | | | | |
| PreCG.L | | | 0.51 | **0.0003** | 0.18 | 0.3340 |  | 0.16 | 0.4111 |
| PreCG.R^1^ | | | 0.41 | **0.0042** | 0.06 | 0.7506 |  | 0.25 | 0.1865 |
| pOp.L | | | 0.17 | 0.2534 | -0.23 | 0.2069 |  | 0.31 | 0.0986 |
| MOG.L | | | 0.45 | **0.0015** | 0.27 | 0.1359 |  | -0.01 | 0.9464 |
| pTr.R | | | 0.37 | **0.0098** | -0.17 | 0.3501 |  | 0.47 | **0.0108** |
| PreC.R | | | 0.39 | **0.0075** | 0.08 | 0.6515 |  | 0.06 | 0.7571 |
| ITG.L | | | 0.39 | **0.0064** | 0.03 | 0.8703 |  | 0.07 | 0.7281 |
| PreCG.R^2^ | | | 0.39 | **0.0061** | -0.07 | 0.7237 |  | 0.13 | 0.5089 |
| MFG.R | | | 0.44 | **0.0019** | 0.10 | 0.5800 |  | 0.28 | 0.1473 |
| **Phonemic task** | | | | | | | | | |
| IPL.L^1^ | | | 0.36 | **0.0125** | 0.29 | 0.1156 |  | -0.07 | 0.7335 |
| PreCG.L | | | 0.36 | **0.0140** | 0.09 | 0.6200 |  | -0.03 | 0.8969 |
| IPL.L^2^ | | | 0.27 | 0.0686 | 0.13 | 0.4859 |  | 0.12 | 0.5452 |
| pTr.R | | | 0.32 | **0.0266** | 0.09 | 0.6320 |  | 0.38 | **0.0393** |
| IOG.L | | | 0.25 | 0.0941 | 0.18 | 0.3260 |  | -0.05 | 0.7935 |
|  |  |  | | | | | | | |

Abbreviations: L, left; R, right; PreCG, precentral gyrus; pOp, pars opercularis; pTr, pars triangularis; MOG, middle occipital gyrus; PreC, precuneus; ITG, inferior temporal gyrus; MFG, middle frontal gyrus; IPL, inferior parietal lobule; IOG, inferior occipital gyrus. ^1, 2^ Clusters in the same anatomical region.

**Supplementary Table 8**. Post hoc analysis of cluster activation. Bold indicated significance (Bonferroni corrected).

|  | **One-way ANOVA** | | **NC v.s. aMCI** | **NC v.s. AD** | **aMCI v.s. AD** |
| --- | --- | --- | --- | --- | --- |
| **Contrast** | **F** | ***P*** |  |  |  |
| **Semantic task** | | | | | |
| PreCG.L | 20.77 | <0.000 | **t = 5.253, *P* < 0.001** | **t = 6.558, *P* < 0.001** | t = 0.844, *P* = 0.406 |
| PreCG.R | 14.20 | <0.000 | **t = 5.249, *P* < 0.001** | **t = 3.980, *P* < 0.001** | t = -1.063, *P* = 0.297 |
| pOp.L | 12.23 | <0.000 | **t = 5.234, *P* < 0.001** | t = 1.508, *P* = 0.142 | **t = -2.929, *P* = 0.007** |
| MOG.L | 18.70 | <0.000 | **t = 2.699, *P* = 0.011** | **t = 6.356, *P* < 0.001** | **t = 3.154, *P* = 0.004** |
| pTr.R | 13.34 | <0.000 | **t = 5.422, *P* < 0.001** | **t = 3.584, *P* = 0.001** | t = -1.677, *P* = 0.104 |
| PreC.R | 14.86 | <0.000 | **t = 5.314, *P* < 0.001** | **t = 3.948, *P* < 0.001** | t = -1.448, *P* = 0.159 |
| ITG.L | 16.93 | <0.000 | t = 2.550, *P* = 0.016 | **t = 5.853, *P* < 0.001** | **t = 3.059, *P* = 0.005** |
| PreCG.R | 13.84 | <0.000 | **t = 5.191, *P* < 0.001** | **t = 4.368, *P* < 0.001** | t = -0.413, *P* = 0.682 |
| MFG.R | 10.69 | <0.000 | **t = 3.979, *P* < 0.001** | **t = 4.687, *P* < 0.001** | t = 0.296, *P* = 0.769 |
| **Phonemic task** | | | | | |
| IPL.L | 15.74 | <0.000 | **t = 5.521, *P* < 0.001** | **t = 4.751, *P* < 0.001** | t = 0.335, *P* = 0.740 |
| PreCG.L | 22.84 | <0.000 | **t = 4.443, *P* < 0.001** | **t = 7.367, *P* < 0.001** | t = 1.964, *P* = 0.059 |
| IPL.L | 13.26 | <0.000 | **t = 4.520, *P* < 0.001** | **t = 4.693, *P* < 0.001** | t = 0.798, *P* = 0.431 |
| pTr.R | 12.75 | <0.000 | **t = 5.304, *P* < 0.001** | **t = 3.548, *P* = 0.001** | t = -1.595, *P* = 0.122 |
| IOG.L | 18.61 | <0.000 | t = 1.614, *P* = 0.117 | **t = 5.699, *P* < 0.001** | **t = 4.206, *P* < 0.001** |
|  | | | | | |
